# Appendiceal goblet cell adenocarcinoma with perineural invasion extending into the ileocecal lesion

**DOI:** 10.1186/s40792-024-01984-w

**Published:** 2024-08-07

**Authors:** Yuka Hosokawa, Sunao Fujiyoshi, Ken Imaizumi, Kengo Shibata, Nobuki Ichikawa, Tadashi Yoshida, Shigenori Homma, Takeaki Kudo, Nanase Okazaki, Utano Tomaru, Akinobu Taketomi

**Affiliations:** 1 Department of Gastroenterological Surgery 1, Hokkaido University Hospital, N-15 W-7, Kita-Ku, Sapporo, Hokkaido 060-8638 Japan; 2 Hamanasu Hospital, 4-1-141-1, Bannaguro, Ishikari, Hokkaido 061-3284 Japan; 3 Department of Surgical Pathology, Hokkaido University Hospital, N-15 W-7, Kita-Ku, Sapporo, Hokkaido 060-8638 Japan

**Keywords:** Goblet cell adenocarcinoma, Appendix, Perineural extension

## Abstract

**Background:**

Appendiceal goblet cell adenocarcinoma (GCA) is a rare subtype of primary appendiceal adenocarcinoma with an incidence of 1–5 per 10,000,000 people per year. Appendiceal tumors are often diagnosed after appendectomy for acute appendicitis. Notably, however, there is currently no standard treatment strategy for GCA, including additional resection. We report a case of appendiceal GCA with perineural extension into the cecum, in which ileal resection was considered effective.

**Case presentation:**

A 41-year-old man was diagnosed with acute appendicitis and underwent appendectomy. Histopathological findings revealed GCA (T3, Pn1). He was referred to our hospital for additional resection. Preoperative examination indicated a diagnosis of GCA cT3N0M0. Laparoscopic ileocecal resection and D3 lymph node dissection were performed 2 months after initial appendectomy. The patient had a good postoperative course and was discharged 8 days after surgery. Histopathological findings showed a GCA invading the cecum, despite an intact appendiceal stump, no lymph node metastasis, no vascular invasion, and no horizontal extension into the submucosa. Direct invasion of the tumor through the serosa was not observed, but perineural extension was conspicuous in the cecum, suggesting that the GCA extended into the cecum via perineural invasion. The resection margins were negative. The patient has survived free of recurrence for a year after ileocecal resection.

**Conclusions:**

The current patient was diagnosed with appendiceal GCA following appendectomy for acute appendicitis. Despite intact of appendiceal stump and no evidence of lymph node or distant metastasis, he underwent laparoscopic ileocecal resection and D3 lymph node dissection 2 months after initial appendectomy, with a favorable outcome. Despite the detection of perineural invasion, the patient declined adjuvant therapy. This case suggests that extensive resection may be required in patients with appendiceal GCA, but the role of adjuvant therapy remains unclear.

## Background

Appendiceal goblet cell adenocarcinoma (GCA) is a rare subtype of primary appendiceal adenocarcinoma with an incidence of 1–5 per 10,000,000 people per year. GCA accounts for approximately 15% of all appendiceal neoplasms [1, 2]. Appendiceal tumors are difficult to diagnose preoperatively and are often diagnosed incidentally after appendectomy for acute appendicitis [3]. In addition, treatment strategies for GCA, such as additional resection, are not well defined. We report a case of appendiceal GCA with perineural extension into the cecum, in which ileal resection was considered effective.

## Case presentation

**Fig. 1:**
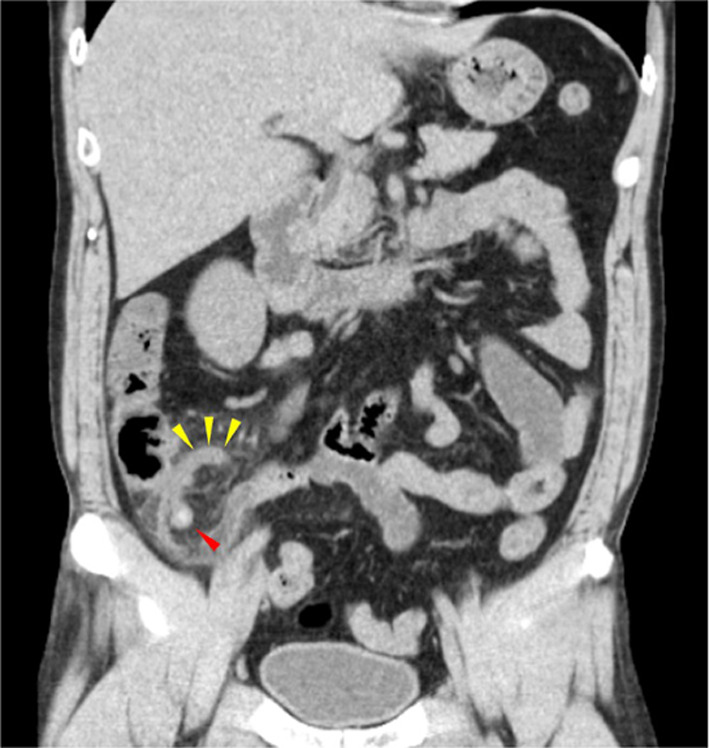
Abdominal computed tomography (CT) images showing enlarged appendix (yellow arrowheads) with periappendiceal fat stranding. A fecal stone (red arrowhead) was observed at the root of the appendix

A 41-year-old man presented with right lower quadrant abdominal pain. One day prior to presentation, he developed right lower abdominal pain, which worsened and was examined by his previous physician. Laboratory evaluation revealed leukocytosis (21,200/μL) and increased C-reactive protein levels (6.00 mg/dL). A computed tomography (CT) scan revealed an enlarged appendix with periappendiceal fat stranding. A fecal stone was observed at the root of the appendix (Fig. 1). He was diagnosed with acute appendicitis and underwent appendectomy at his previous hospital.

**Fig. 2:**
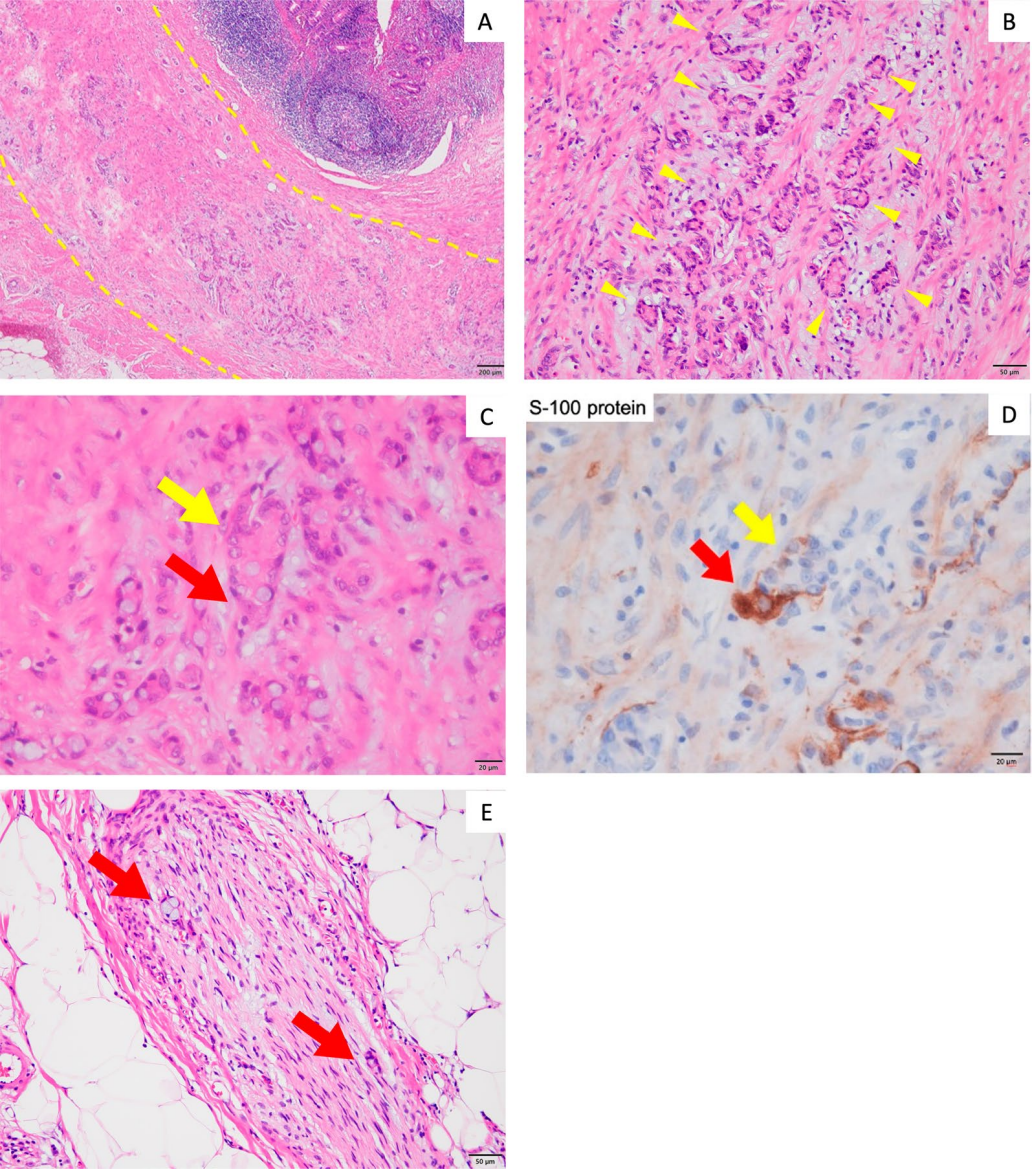
Histopathological findings showing goblet cell adenocarcinoma, Grade 1. **A** Tumor cells (yellow dashed line) were generally confined to the muscularis propria (hematoxylin and eosin (H&E) staining, ×40 magnification). **B** Most of the tumor cells (yellow arrowheads) showed a low-grade pattern of clustered growth (H&E staining, ×200 magnification). **C** Tumor cell clusters (yellow arrow) adjacent to ganglion cells with well-defined nucleolus (red arrow), (H&E staining, ×400 magnification). **D** Tumor cell cluster (yellow arrow) adjacent to S-100 protein-positive ganglion cell (red arrow), indicating perineural invasion (S-100 staining, ×400 magnification). **E** Tumor cells extend extra-mural within perineural invasion (H&E staining, ×400 magnification)

The histopathologic diagnosis was GCA. Most of the tumor showed a low-grade pattern of tubular or clustered growth and was classified as Grade 1 according to the World Health Organization (WHO) Classification of Tumours, 5th edition [4] (Fig. 2A). The tumor was generally confined to the muscularis propria (Fig. 2B). HE staining showed the tumor cells in contact with ganglion cells with well-defined nucleolus (Fig. 2C). Staining for the neuron-specific marker S-100 protein also showed that the ganglion cells were in contact with tumor vesicles (Fig. 2D). Even though the appendiceal stump was intact and there was no tumor exposure to the serosal surface, perineural invasion (PNI) was scattered in the subserosa and appendiceal mesentery, which suggests that the tumor had extended extra-mural by perineural invasion (Fig. 2E). The postoperative diagnosis was appendiceal GCA pT3, Pn1, cN0, cM0, pStage IIA (Union for International Cancer Control (UICC); 8th edition, 2017). He was referred to our hospital for close examination and treatment.

The patient had a history of hypertension but was not taking any medications. He had no allergies. He had drunk distilled spirits daily for 21 years and had a smoking history of 40 cigarettes per day for 23 years. His father had colon cancer. On physical examination, he was 180 cm tall, weighed 90 kg, and his body mass index was 27.8. The abdomen was flat and soft, with no tenderness.

**Fig. 3:**
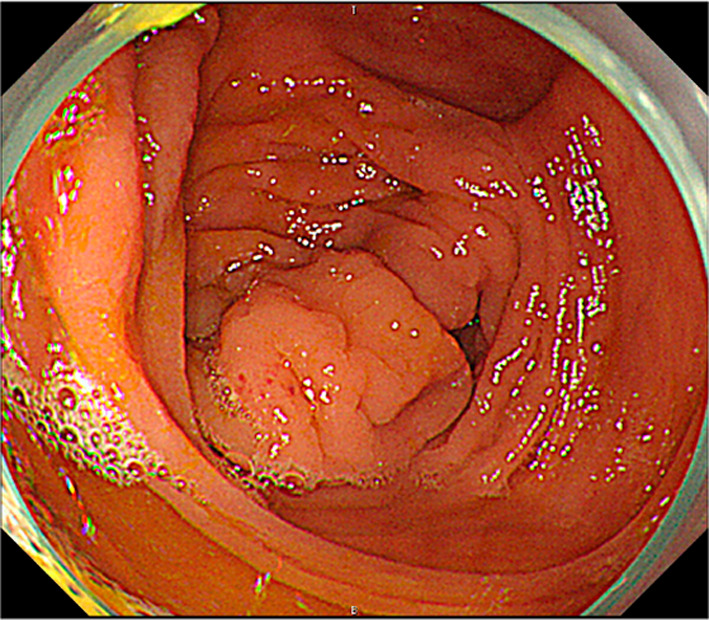
Colonoscopy revealed no mass in the cecum

**Fig. 4:**
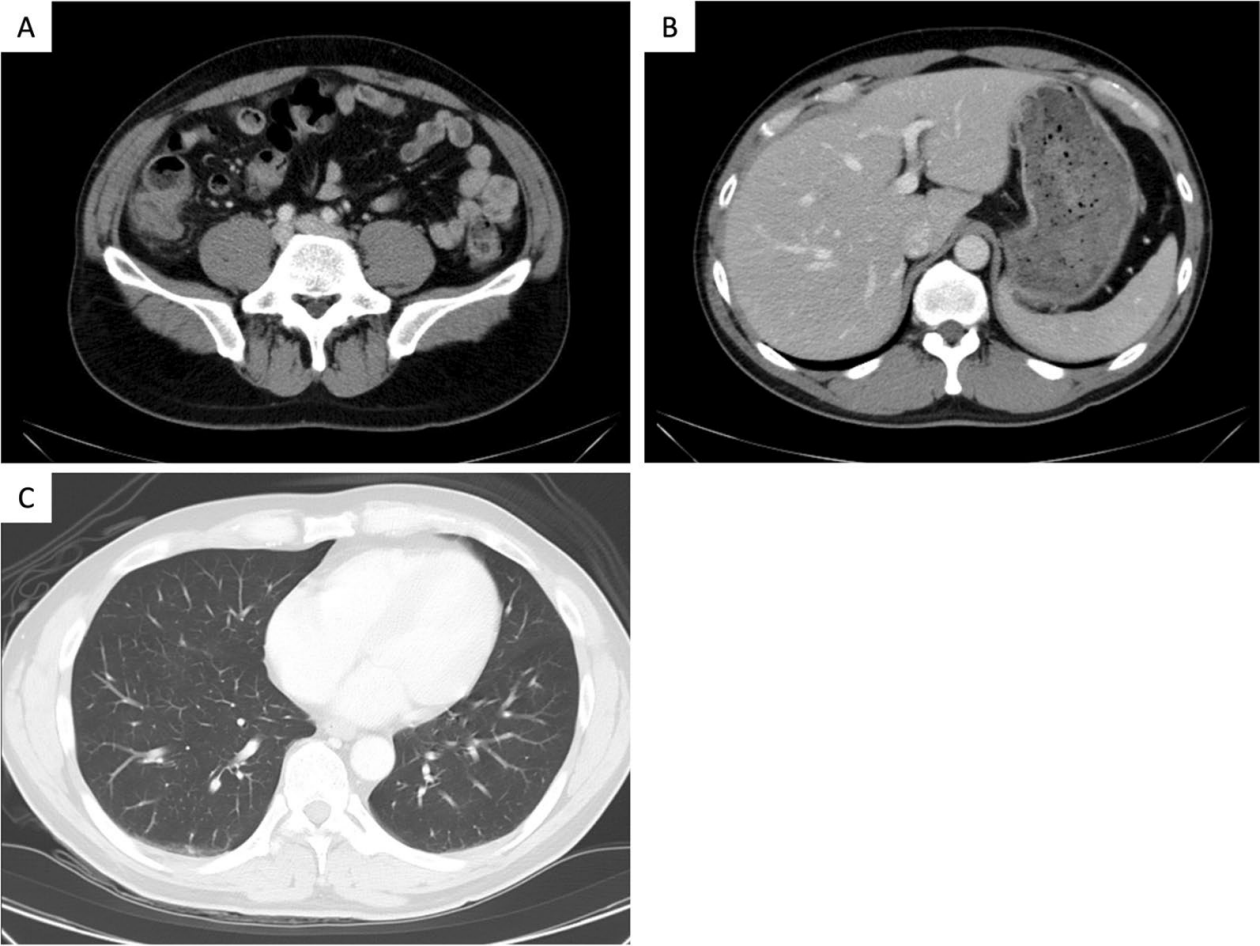
Preoperative CT showed no mass or enlarged lymph nodes.** a** There is not mass in the ileocecal area.** b** There is not hepatic mass.** c** There is not lung mass.

Laboratory evaluation revealed no specific findings and no elevated tumor markers (Table 1). Colonoscopy showed no mass in the cecum at the appendiceal orifice (Fig. 3). CT scan revealed no mass lesions in the ileocecum after appendectomy and no lymph node or distant metastasis (Fig. 4). The preoperative diagnosis was appendiceal GCA cT3N0M0 Stage IIa, and additional resection was considered as for an appendiceal tumor.

**Table 1:** Preoperative laboratory data

Hematology			Biochemistry		
WBC	5800		TP	7.2	g/dL
Hb	15.9	g/dL	Alb	4.5	g/dL
Plt	23.8 × 10^4^	/μL	T-Bil	0.4	mg/dL
			AST	20	U/L
Coagulation			ALT	27	U/L
PT-INR	0.92		γ-GTP	59	U/L
APTT	27.4	s	ALP	63	U/L
Fib	323	μg/mL	LD	146	U/L
			BUN	13	mg/dL
Tumor marker			Cr	0.84	mg/dL
CEA	2.8	ng/mL	Na	141	mEq/L
CA19-9	25.7	U/mL	K	5.0	mEq/L
			Cl	104	mEq/L
			CRP	0.03	mg/dL
			HbA1c	5.9	%

*WBC* white blood cells, *Hb* hemoglobin, *Plt* platelet, *PT-INR* prothrombin time-international normalized ratio, *APTT* activated partial thromboplastin time, *Fib* fibrinogen, *CEA* carcinoembryonic antigen, *CA19-9* carbohydrate antigen 19-9, TP total protein, *Alb* albumin, *T-Bil* total bilirubin, *AST* aspartate aminotransferase, *ALT* alanine aminotransferase, *γ-GTP* γ-glutamyl transpeptidase, *ALP* alkaline phosphatase, *LD* lactate dehydrogenase, *BUN* blood urea nitrogen, *Cr* creatinine, *Na* sodium, *K* potassium, *Cl* chlorine, *CRP* C-reactive protein, *HbA1c* hemoglobin A1c

**Fig. 5:**
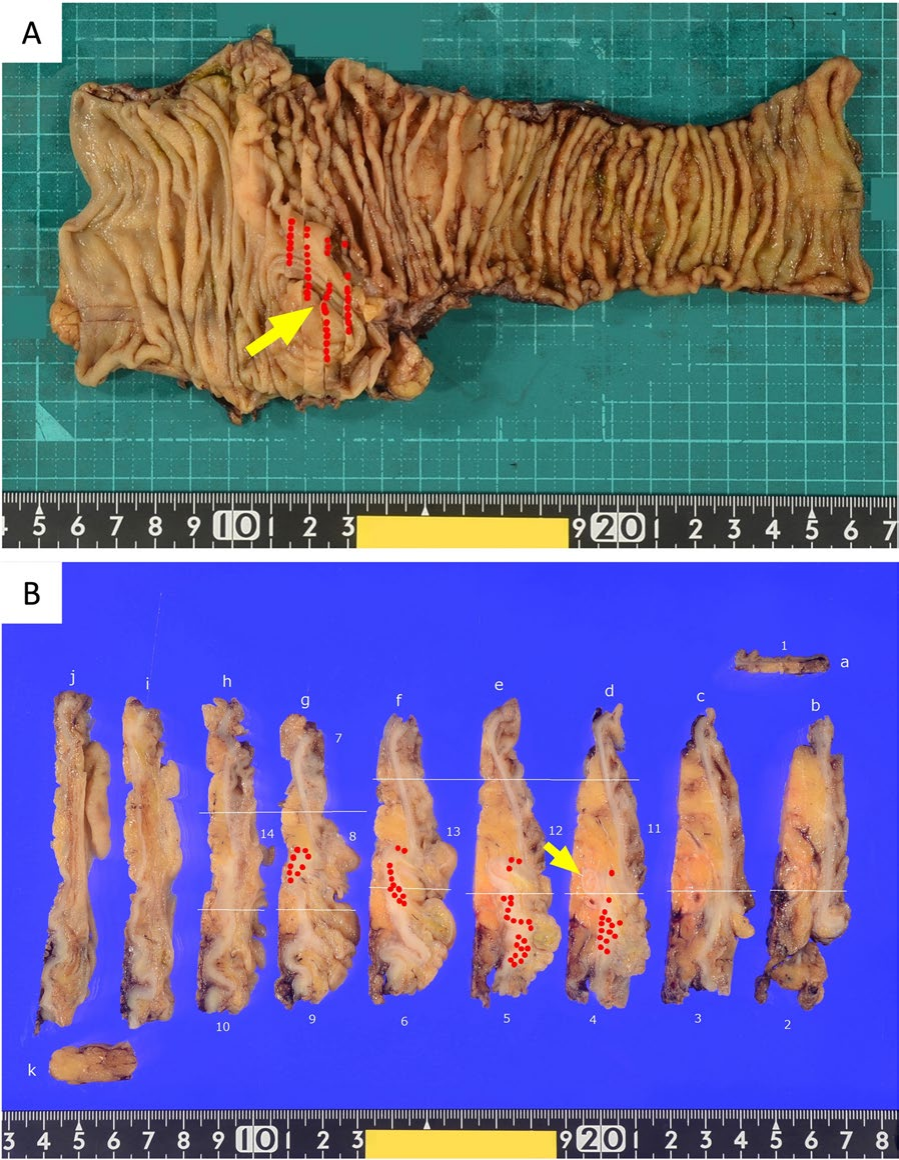
**A** Tumor mapping showed goblet-like cells extending discontinuously into the ileocecum (red dots: tumor location), (yellow arrow: appendix orifice). **B** Tumor mapping of cross section showed that the appendiceal orifice was intact appendiceal orifice (red dots: tumor location), (yellow arrow: appendix orifice)

**Fig. 6:**
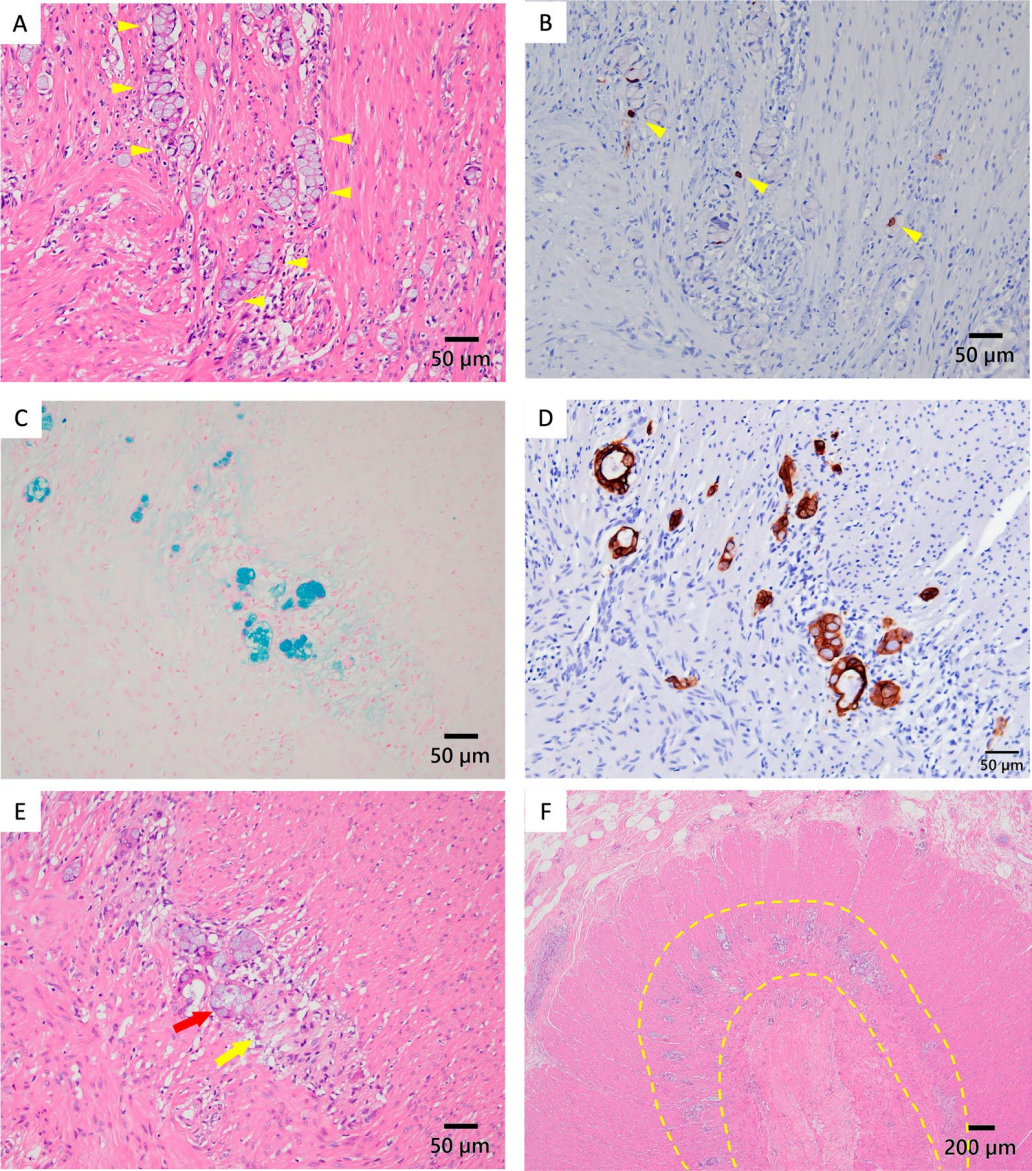
Immunohistochemistry and histochemistry findings. **A** Some tumor cells clustered as small groups of cohesive goblet-like cells with a low-grade pattern (H&E staining, yellow arrowheads, ×400 magnification). **B** Tumor cells weakly positive for the neuroendocrine marker chromogranin A (yellow arrowheads, ×400 magnification). **C** Tumor cells observed as goblet cells with mucus production (Alcian blue stain, ×400 magnification). **D** Tumor cells positive for the epithelial marker cytokeratin AE1/AE3 in the muscularis propria of the cecum (×400 magnification). **E** Tumor cell clusters (red arrow) are in contact with nerves (yellow arrow), (H&E staining, ×40 magnification). **F** Tumor cells (yellow dashed line) distributed intramural along the Auerbach plexus area (H&E staining, ×40 magnification)

Laparoscopic ileocecal resection with D3 lymph node dissection was performed on day 58 after appendectomy, in accordance with the JSCCR Guidelines 2024 for the Treatment of Colorectal Cancer. No gross mass was detected in the resection specimen. Histopathology revealed goblet-like cells extending discontinuously into the ileocecum, despite an intact appendiceal stump (Fig. 5A, B). The distance between appendix orifice and the closest part of the cecum tumor was 2.5 mm. Hematoxylin and eosin staining showed some tumor cells clustered as small groups of cohesive goblet-like cells with a low-grade pattern (Fig. 6A). Staining for the neuroendocrine marker, chromogranin A, was weakly positive (Fig. 6B). Alcian blue staining was positive, indicating mucus production by tumor cells (Fig. 6C). The tumor cells in the muscularis propria of the cecum were also positive for the epithelial marker cytokeratin AE1/AE3 (Fig. 6D). The lesions were generally confined to the intrinsic muscularis propria and did not appear to involve the serosal surfaces with no evidence of lymphatic or venous invasion. However, the tumor cells were adjacent to the nerve and distributed within the muscularis propria of the cecum along the Auerbach plexus area (Fig. 6E, F), suggesting that the GCA extended into the cecum by PNI [5, 6]. No malignant cells were found at the resection margins, and there was no lymph node metastasis. The final diagnosis was GCA, pathological stage pT3pN0M0 pStage IIA (UICC 8th edition).

The patient had a good postoperative course and was discharged 8 days after surgery. He remained under observation. The patient has survived free of recurrence for a year after ileocecal resection. The patient did not receive postoperative adjuvant chemotherapy, partly because there is no evidence of its efficacy in Stage II tumors and because he declined the option.

## Discussion

GCA was first recognized by Gagne in 1969 as a tumor of the appendix with histologic carcinoid and adenocarcinoma features [7]. Its classification is currently not defined, and GCA is, therefore, known by several different names [8]. The 4th edition of the WHO Classification of Digestive System Tumors referred to GCA as goblet cell carcinoid and classified it as an appendiceal endocrine tumor and a subtype of mixed adeno-neuroendocrine carcinoma; however, GCAs are more aggressive than typical well-differentiated neuroendocrine tumors of the appendix. The 5th edition of the WHO Classification of Digestive System Tumors gives GCA as the preferred diagnosis, because of the increasing recognition of a frequent co-existing high-grade adenocarcinoma component, and it was classified as a subtype of adenocarcinoma in 2019 [9]. The ambiguous disease classification, however, means that no treatment strategy for GCA has yet been established.

GCA is not usually mass-forming, and the appendix may appear grossly unremarkable [10], and unlike other appendiceal diseases, the CT imaging findings are variable. GCA is, therefore, difficult to identify preoperatively and most cases are diagnosed incidentally after appendectomy. Histologically, appendiceal GCA is an amphicrine tumor composed of goblet-like mucinous cells, as well as variable numbers of endocrine cells and Paneth-like cells, typically arranged as tubules resembling intestinal crypts [11]. All GCAs stain positive with periodic acid-Schiff and Alcian blue for mucin, while immunohistochemical staining shows pronounced expression of the neuroendocrine markers chromogranin A and synaptophysin [12]. In the present case, the tumor grew as a cluster composed of goblet-like mucinous cells with a small number of mixed endocrine cells, leading to the diagnosis of GCA.

The most recent tumor, node, metastasis (TNM) staging system of the combined American Joint Committee on Cancer and UICC classification stages mixed-histology tumors such as GCA using the staging classification for appendiceal carcinomas. The reported 5-year survival rates for GCA Stages I, II, III, and IV are 91.1%, 90.5%, 57.0%, and 18.9%, respectively [13]. The rate of lymph node positivity increases in a stepwise fashion, with lymph node-positivity rates of 1.1%, 2.1%, 9.9%, and 29.1% for T1, T2, T3, and T4 tumors, respectively [14].

The growth pattern of GCA is within the lamina propria and extends through the muscularis propria into the subsequent serosa, with the mucosa remaining intact [15]. GCA has the potential to spread intraperitoneally, even in the absence of nodal metastases [16]. Mcbey et al. showed that only 17% of 224 patients who underwent right hemicolectomy had lymph node metastasis, but 65% of them showed spread through the serosa, invasion of the mesoappendix, or extension to adjacent organs or peritoneum [1]. GCA rarely metastasizes hematogenously to the liver and lungs, despite being widely disseminated on the abdominal surfaces [17], but PNI has been reported in approximately 25% of GCAs and is common among all grades [11, 17]. One study found that PNI was associated with a poor prognosis in multivariate analysis [17]. PNI is often described as skip lesions, which are thought to indicate neoplastic infiltration of a nerve with areas of disease-free nerve [18]; however, some pathologist argue that skip lesions are merely a processing artifact [19]. The existence of skip lesions in cases of PNI makes it difficult to determine the pathological resection margin, leading to the possibility of residual tumor. Thus, even if the appendiceal stump is negative but PNI is positive, as in the current case, additional resection is considered necessary because of the possibility of extension into the cecum.

The most important treatment for GCA is curative resection of the tumor. The American Society of Colon and Rectal Surgeons, the North American Neuroendocrine Tumor Society, and the European Neuroendocrine Tumor Society all recommend right hemicolectomy as the standard surgical treatment for appendiceal GCA of any stage or histology [20–22]. In Japan, however, there is currently no consensus on the optimal management strategy for GCA due to the rarity of the disease. It has been reported that 38% of tumors were upstaged following secondary right hemicolectomy, as a result of invasion of lymph nodes in the mesentery [23]. Although lymph node metastasis was negative in the present case, lymph node dissection should be performed because GCA has a high incidence of potential lymph node metastasis to the mesentery.

A German multicenter series reported recurrence rates of 0%–12%, 21%–41%, and 61%–75% for Stages I, II, and III appendiceal carcinoma, respectively [23–25], mostly with peritoneal recurrence [26]. Appendiceal neoplasms are frequently associated with perforated appendicitis and the tumor cells are thus likely to reach the serosa because of the thin muscular propria [27]. The value of adjuvant chemotherapy remains unclear, but some reports showed that adjuvant treatment, as for adenocarcinoma, prolonged overall survival in patients with node-positive (Stage III) disease [13, 14]. Although the current patient had Stage II disease, PNI was observed and the risk of recurrence was thus considered to be high, and we, therefore, proposed adjuvant therapy; however, the patient refused because of the risk of adverse events. Adjuvant therapy may help to control peritoneal recurrence and prolong overall survival; however, further studies are needed to investigate the indications for adjuvant therapy.

## Conclusions

Appendiceal GCA has a relatively poor prognosis. Appendectomy may not be sufficient, even in patients with no preoperative evidence of distant metastases or lymph node involvement, or with negative resection margins, and especially in cases with PNI. Additional ileocecal resection, similar in extent to the resection of the adenocarcinoma, may be necessary because of the possibility of tumor invasion. Further studies are needed to determine the strategy in relation to adjuvant therapy.

## Data Availability

All the data generated or analyzed during this study are included in this article.

## Abbreviations

CT: Computed tomography
GCA: Goblet cell adenocarcinoma
PNI: Perineural invasion
